# Analysis of Lymph Node Metastasis Correlation with Prognosis in Patients with T2 Gastric Cancer

**DOI:** 10.1371/journal.pone.0105112

**Published:** 2014-08-19

**Authors:** Xiaowen Liu, Ziwen Long, Hong Cai, Hua Huang, Yingqiang Shi, Yanong Wang

**Affiliations:** 1 Department of Gastric Cancer and Soft Tissue Sarcoma, Fudan University Shanghai Cancer Center, Shanghai, China; 2 Department of Oncology, Shanghai Medical College, Fudan University, Shanghai, China; University of North Carolina School of Medicine, United States of America

## Abstract

**Purpose:**

To investigate the correlated factors for lymph node metastasis and prognosis for patients with T2 gastric cancer.

**Methods:**

A total of 442 patients with T2 gastric cancer who underwent gastrectomy from January 1996 to December 2009 were evaluated. The clinicopathological parameters were analyzed for lymph node metastasis and prognosis, including gender, age, tumor size, tumor location, histological type, depth of invasion, vascular tumor emboli, nervous invasion, resection type, and pathological stage.

**Results:**

The rate of lymph node metastasis was 45.9%. Univariate analysis showed that depth of invasion, tumor size, and vascular tumor emboli were associated with lymph node metastasis. Logistic regression demonstrated that depth of invasion, tumor size, and vascular tumor emboli were independently predictive factors for lymph node metastasis. The 5-year survival rate was 64.0%. Multivariate analysis showed that tumor size, tumor location, resection type, and pathological stage were independent prognostic factors. Based on tumor size, there were significant differences of 5-year survival between small size tumor (<6 cm) and large size tumor (≥6 cm) according to stage IIA (*P* = 0.006). Based on tumor location, there were significant differences of 5-year survival among different tumor location according to stage IB. Based on resection type, there were significant differences of overall 5-year survival between curative surgery and palliative surgery according to stage IIB (*P* = 0.015) and IIIA (*P* = 0.001).

**Conclusion:**

Depth of invasion, tumor size, and vascular tumor emboli were independently predictive factors for lymph node metastasis. Tumor size, tumor location, resection type, and pathological stage were independent prognostic factors.

## Introduction

Although the incidence of gastric cancer has been dramatically declining for past several decades, it remains the fourth most common cancer and the second most frequent cause of cancer-related death worldwide [1], [2]. The identification of prognostic factors is essential for predicting patients' survival and determining optimal therapeutic strategies. A variety of prognostic factors including pathological parameters and biological markers have been reported [3]–[6]. Among the prognostic factors now available for gastric cancer, the most precise and important prognostic factor still was the UICC TNM stage. Some studies have indicated that the depth of invasion and the status of lymph node metastasis were the most important prognostic factors in gastric cancer [7], [8].

The presence or absence of lymph node metastasis was closely related with depth of invasion [9]. The rate of lymph node metastasis was relatively low in T1 stage gastric cancer, the rate of lymph node metastasis was relatively high in T3 or more stage gastric cancer. However, there is a large variance in patients with T2 stage gastric cancer. Additionally, there was a significant modification in T2 stage between the sixth and seventh UICC/AJCC TNM classification. In the former, the T2 stage indicated that tumor extended into the muscularis propria (MP) or subserosa (SS) layer. In the latter, the tumor invading into subserosa (SS) was classified into T3 stage. There were relatively limited studies reporting the clinicopathological characteristics and prognosis of patients with T2 stage gastric cancer in the seventh UICC stage system. Therefore, we evaluated the correlated factors for lymph node metastasis and prognosis in patients with T2 gastric cancer.

## Materials and Methods

### Patients

From January 1996 to December 2009, 4550 patients with histologically confirmed primary gastric adenocarcinoma underwent gastrectomy at the Department of gastric cancer and soft sarcoma, Fudan University Shanghai Cancer Center. Inclusion criteria for this study were as follows: (1) tumor invading into but not penetrating muscularis propria; (2) complete pathological data. There were 477 patients presented with invasion of the muscularis propria (T2). Of 477 patients, 35 were excluded from the final analysis: 13 received preoperative chemotherapy; five due to gastric stump carcinoma; 17 had distant metastasis. In total, 442 patients were included in the current study. Data were retrieved from their operative and pathological reports, and follow-up data were obtained by phone outpatient and clinical database. The written informed consent had been obtained from all the patients, and this study was approved by the Ethical Committee of Shanghai Cancer Center of Fudan University. The study was retrospective. Patient records were anonymized and de-identified prior to analysis.

### Preoperative evaluation and treatment

Imaging studies were routinely performed using upper gastrointestinal barium-meal, endoscopic examination, and computed tomographic scan in order to evaluate the preoperative stage. Staging was performed according to the American Joint Committee on Cancer (AJCC) TNM Staging Classification for Carcinoma of the Stomach (Seventh Edition, 2010). Gastrectomy was performed in accordance with the Japanese Classification of Gastric Carcinoma.

### Follow-up

A follow-up of all patients was carried out according to our standard protocol (every three months for at least 2 years, every six months for the next 3 years, and after 5 years every 12 months for life). The check-up items included physical examination, tumor-marker examination, ultrasound, chest radiography, computed tomographic scan, and endoscopic examination. The median follow-up time was 70 months for all patients.

### Statistical analysis

The patients' features and clinicopathological characteristics were analyzed using the two-tailed Student's *t*-test for continuous variable and χ^2^ test for categorical variable. Logistic regression was used to estimate related factors for lymph node metastasis. Five-year survival rate was calculated by Kaplan-Meier method, and the differences between survival curves were examined with long-rank test. Factors that were deemed of potential importance on univariate analysis were included in the multivariate analysis. The independent prognostic factors were examined by the multivariate survival analysis using Cox proportional hazards model. The accepted level of significance was *P*<0.05. Statistical analyses and graphics were performed using the SPSS 13.0 statistical package (SPSS, Inc., Chicago, IL).

## Results

### Clinicopathological characteristics

There were 302 males and 140 females (2.2∶1) with a mean age of 57 years. There were 263 (59.5%) patients with infiltration in shallow muscular (T2a), and 179 (40.5%) patients with infiltration in deep muscular invasion (T2b). According to histological type, well-differentiated tumors were observed in 13 (2.9%) patients, moderately-differentiated in 129 (29.2%) patients, poorly-differentiated tumors in 281 (63.6%) patients, and undifferentiated tumors remaining 19 (4.3%). Of these patients, 101 (22.9%) had tumors located in the upper third (70 patients with Siewert II, 31 patients with Siewert III), 65 (14.7%) had tumors in the middle third, 253 (57.2%) had tumors in the lower third of the stomach, and 23 (5.2%) had tumors occupied two-thirds or more of stomach. Total number of lymph nodes dissected was 7395 (mean 16.7, median 16.0). Lymph node metastasis was observed in 203 patients, the metastasis rate was 45.9%. There were 28 patients receiving palliative resection. The reason for palliative resection was that the standard D2 lymph node dissection was not enough for these patients with T2N3M0. There were 216 patients received adjuvant chemotherapy. The demographic data were shown in Table 1.

**Table 1:** Clinicopathologic characteristics of patients.

Clinicopathologic features	Number (%)
Gender	
Female	140 (31.7)
Male	302 (68.3)
Age (years)	
≤50	113 (25.6)
>50	329 (74.4)
Tumor size (cm)	
<6	390 (88.2)
≥6	52 (11.8)
Tumor location	
Upper third	101 (22.9)
Middle third	65 (14.7)
Lower third	253 (57.2)
≥Two thirds	23 (5.2)
Histological type	
Well-differentiated	13 (2.9)
Moderately-differentiated	129 (29.2)
Poorly-differentiated	281 (63.6)
Undifferentiated	19 (4.3)
Depth of invasion in muscularis propria	
Shallow muscular layer	263 (59.5)
Deep muscular layer	179 (40.5)
Vascular tumor emboli	
Yes	105 (23.8)
No	337 (76.2)
Nervous invasion	
Yes	67 (15.2)
No	375 (84.8)
Lymph node metastasis	
Yes	203 (45.9)
No	239 (54.1)
Pathological stage	
IB (T2N0M0)	239 (54.1)
IIA (T2N1M0)	91 (20.6)
IIB (T2N2M0)	65 (14.7)
IIIA (T2N3M0)	47 (10.6)

### Risk factors for lymph node metastasis

Of the 442 patients with invasion in muscularis propria, 203 patients (45.9%) had lymph node metastasis. Univariate analysis showed that depth of invasion, tumor size, vascular tumor emboli were associated with lymph node metastasis, whereas age, gender, histological type, tumor location, and nervous invasion were not (Table 2). Logistic regression was employed to identify the independent risk factors of lymph node metastasis. It was demonstrated that depth of invasion, tumor size, and vascular tumor emboli were independently predictive factors for lymph node metastasis (Table 3).

**Table 2:** Univariate analysis of predictors of lymph node metastasis.

Variable	No metastasis	Metastasis	P
	(n = 239)	(n = 203)	
Gender			0.335
Male	168	134	
Female	71	69	
Age (years)			0.526
≤50	64	49	
>50	175	154	
Tumor size (cm)			0.007
<6	220	170	
≥6	19	33	
Tumor location			0.662
Upper third	56	45	
Middle third	39	26	
Lower third	133	120	
Two or more thirds	11	12	
Histological type			0.147
Well differentiated	10	3	
Moderate differentiated	72	57	
Poor differentiated	144	137	
Undifferentiated	13	6	
Depth of invasion in muscularis propria			0.022
Shallow layer	154	109	
Deep layer	85	94	
Vascular tumor emboli			0.000
Yes	29	76	
No	210	127	
Nervous invasion			0.097
Yes	30	37	
No	209	166	

**Table 3:** Multivariate analysis of predictors of lymph node metastasis.

Variable	Hazard ratio	95% confidence interval	P value
Age	1.040	0.660–1.641	0.865
Tumor size	1.968	1.043–3.713	0.037
Vascular tumor emboli	4.056	2.493–6.599	0.000
Depth of invasion	1.517	1.013–2.271	0.043

### Univariate analysis

The over-all 5-year survival rate was 64.0% for all 442 patients. The significant prognostic factors included: tumor size, tumor location, depth of invasion, lymph node status, resection type, and pathological stage (Table 4). The 5-year survival was higher in patients with small size tumor (<6 cm) compared with those patients with large size tumor (≥6 cm) (*P* = 0.001). The 5-year survival was longer in patients with lower third location tumor (*P* = 0.032), in patients with shallow muscular invasion (*P* = 0.017), in patients without lymph node metastais (*P* = 0.000), in patients with curative gastrectomy (*P* = 0.000). The survival of patients was better in early stage than that of late stage (*P* = 0.000). The sex, age, histological type, vascular tumor emboli and nervous invasion did not show any relationship with survival.

**Table 4:** Univariate analysis of all patients by Kaplan-Meier method.

Variable	n	5-Year survival rate (%)	*P* value
Gender			0.936
Female	140	63	
Male	302	64	
Age (y)			0.386
≤50	113	61	
>50	329	64	
Tumor size (cm)			0.001
<6	390	66	
≥6	52	47	
Tumor location			0.032
Upper third	101	63	
Middle third	65	57	
Lower third	253	67	
≥Two thirds	23	50	
Histological type			0.134
Well-differentiated		84	
Moderately-differentiated		71	
Poorly-differentiated		59	
Undifferentiated		73	
Depth of invasion			0.017
Shallow muscularis layer	263	66	
Deep muscularis layer	179	60	
Vascular tumor emboli			0.066
Yes	105	55	
No	337	66	
Nervous invasion			0.857
Yes	67	61	
No	375	64	
Lymph node metastasis			0.000
Yes	203	48	
No	239	77	
Curability			0.000
Curative	414	67	
Palliative	28	20	
Pathological stage			0.000
IB (T2N0M0)	239	77	
IIA (T2N1M0)	91	59	
IIB (T2N2M0)	65	42	
IIIA (T2N3M0)	47	35	

### Multivariate analysis

**Figure 1:**
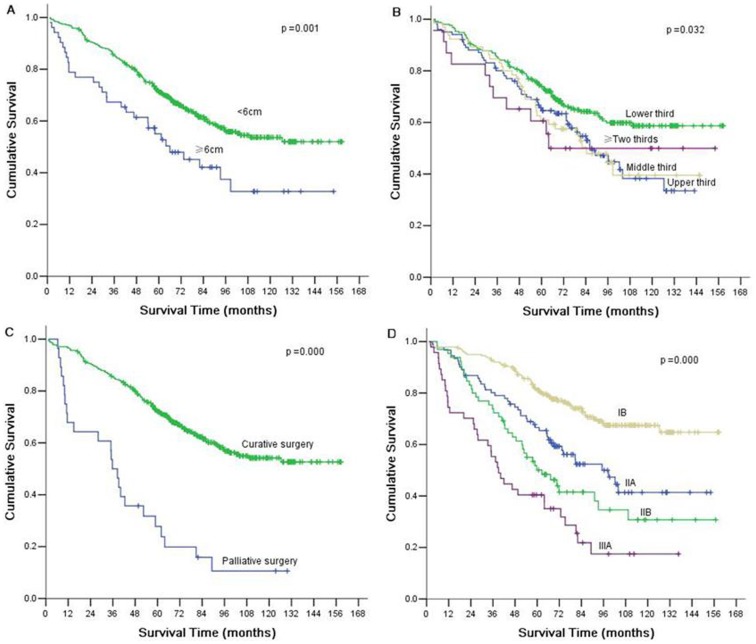
Kaplan-Meier curves for overall survival of T2 gastric cancer according to tumor size (A), tumor location (B), curability (C), and pathological stage (D).

Multivariate survival analysis was performed to determine the independent prognostic factors for T2 gastric cancer. Multivariate analysis using Cox proportional hazards model showed that tumor size, tumor location, resection type, and pathological stage were independent prognostic factors (Table 5) (Figure 1).

**Table 5:** Multivariate analysis of patients by Cox model.

Variable	χ^2^	*P* vale	RR	95% CI
Sex	0.0.173	0.678	0.936	0.684–1.280
Age	0.035	0.851	1.034	0.726–1.473
Tumor size	4.962	0.026	1.574	1.056–2.347
Tumor location	5.906	0.015	0.819	0.697–0.962
Depth of invasion	0.815	0.367	1.148	0.851–1.548
Lymph node status	1.886	0.170	1.443	0.855–2.435
Curability	18.278	0.000	2.719	1.719–4.301
Pathological stage	8.273	0.004	1.396	1.112–1.753

### Comparison of survival according to tumor size, tumor location, and resection type

According to AJCC/TNM Staging System, tumor stage in this study was divided into 4 stages: stage IB, stage IIA, stage IIB, and stage IIIA. Based on tumor size, each stage was divided into small size tumor (<6 cm) and large size tumor (≥6 cm). There were significant differences of over-all 5-year survival between two groups according to stage IIA (*P* = 0.006). Based on tumor location, each stage was divided into upper third, middle third, lower third and two or more third. There were significant differences of over-all 5-year survival among four groups according to stage IB (*P* = 0.040). Based on resection type, each stage was divided into curative surgery and palliative surgery. There were significant differences of over-all 5-year survival among two groups according to stage IIB (*P* = 0.015), IIIA (P = 0.001).

## Discussion

Gastric cancer was one of the most common malignancies worldwide. Although the prognosis of patients with gastric cancer has improved, gastric was still the second leading cause of cancer related deaths as a result of late diagnosis. The identification of prognostic factors is very important for predicting gastric cancer patients' survival and determining therapeutic strategies. It is universally acknowledged that the most significant factors affecting prognosis of gastric cancer patients are the depth of invasion (T staging) and the status of lymph node metastasis (N staging) [10]–[12]. According to the NCCN guideline, the T staging was classified as four categories including T1, T2, T3, and T4. Patients with T1 had a relatively good prognosis; patients with T3 or T4 had poor prognosis. T2 was considered as an intermediate stage between early and advanced gastric cancer. Therefore, there was a large variance in the prognosis of patients with T2 gastric cancer. In this study, the clinicopathological characteristics and prognostic factors were investigated in patients with T2 stage gastric cancer.

The status of lymph node metastasis was considered to be very important prognostic predictor for gastric cancer; patients with lymph node metastasis had a worse survival than those without lymph node metastasis [13]–[15]. Therefore, identification of predictive factors for lymph node metastasis was crucial to establish the staging and prognosis. Although many studies have reported the association between clinicopathological variables and lymph node metastasis in gastric cancer, studies in T2 gastric cancer have been rarely reported so far especially in large-scale. Therefore, we conducted this study in 442 patients with T2 gastric cancer. In this study, we found that depth of invasion, tumor size, vascular tumor emboli were associated with lymph node metastasis. Logistic regression demonstrated that depth of invasion, tumor size, and vascular tumor emboli were independently predictive factors for lymph node metastasis. This was consistent with previous some studies. Yamashita et al. [16] reported that lymphatic involvement, venous involvement, serosal invasion and tumor size were associated with lymph node metastasis in gastric cancer. Lim et al. [17] reported that tumor size, depth of invasion, macroscopic type, and lymphovascular invasion were related to lymph node metastasis in early gastric cancer. Wu et al. [18] reported that poor differentiation, submucosal invasion and large tumor size were independent risk factors for lymph node metastasis in early gastric cancer. All these studies indicated that some pathological parameters such as tumor size, depth of invasion, and vascular tumor emboli could be good predictors for lymph node metastasis. Therefore, we should pay more attention to T2 gastric cancer patients with these adverse factors, and conduct more appropriate treatment plan in clinical practice.

Despite the fact that the most T2 stage patients with lymph node metastasis had a bad prognosis, some of them still had a long term survival. Therefore, the identification of prognostic factors in patients with T2 gastric cancer was the primary purpose of this study. Many factors have been reported to influence the survival of patients with gastric cancer, such as tumor size, depth of invasion, lymphovascular invasion, tumor location, serosal invasion [19]–[23]. However, previous studies did not take into account the effect of the depth of invasion on other clinicopathological parameters. It was difficult to identify the most crucial variables with regard to prognosis because many variables were interrelated. Therefore, precise identification of prognostic factors can be feasible only when the depth of invasion was specified. Recently, Bu et al. [24] showed that TNM stage and lymphatic vascular invasion were independent prognostic factors for T2 gastric cancer patients. In this study, we found that pathological stage, tumor size, tumor location, and resection type were independent prognostic factors. Therefore, we suggested that T2 gastric patients with lymph node metastasis, large size tumor, more than two thirds location, and palliative gastrectomy should receive adjuvant chemotherapy or radiochemotherapy.

To evaluate whether tumor size, tumor location, and resection type can provide additional prognostic information on the basis of TNM stage system, we compared cumulative survival curves according to these independent prognostic factors. The results showed that there were significant differences of over-all 5-year survival between different tumor size groups according to stage IIA; different location groups according to stage IB; different resection groups according to stage IIB and IIIA. These findings indicated that tumor size, tumor location, and resection type could provide additional prognostic information in patients with T2 gastric cancer.

In conclusion, depth of invasion, tumor size, and vascular tumor emboli were independently predictive factors for lymph node metastasis. Tumor size, tumor location, resection type, and pathological stage were independent prognostic factors. These findings were important to develop individualized treatment plans for patients with gastric cancer. T2 gastric cancer patients with large tumor size, upper location, palliative gastrectomy, and late stage should be considered for adjuvant chemotherapy.
